# Structural Behavior of a Semiflexible Polymer Chain in an Array of Nanoposts

**DOI:** 10.3390/polym9080313

**Published:** 2017-07-28

**Authors:** Zuzana Benková, Lucia Rišpanová, Peter Cifra

**Affiliations:** 1 Polymer Institute, Slovak Academy of Sciences, Dúbravská Cesta 9, 845 41 Bratislava, Slovakia; lucia.rispanova@savba.sk (L.R.); Peter.Cifra@savba.sk (P.C.); 2 LAQV@REQUIMTE, Department of Chemistry and Biochemistry, Faculty of Sciences, University of Porto, Rua do Campo Alegre 687, 4168-007 Porto, Portugal

**Keywords:** semiflexible circular chain, array of nanoposts, confinement, DNA, molecular dynamics, microfluidic devices

## Abstract

The structural properties of a flexible and semiflexible circular chain confined in an array of parallel nanoposts with a square lattice cross-sectional projection were studied using coarse-grained molecular dynamics simulations. To address the effect of the circular topology, a comparison with linear analogs was also carried out. In the interpretation of the chain structural properties, the geometry of the post array is considered as a combination of a channel approximating the interstitial volume with the diameter *d*_c_ and a slit approximating the passage aperture with the width *w*_p_. The number of interstitial volumes occupied by a chain monotonically increases with the decreasing ratio *d*_c_/*w*_p_ regardless of the way the geometry of the post array is varied. However, depending on how the array geometry is modified, the chain span along the posts displays a monotonic (constant post separation) or a non-monotonic behavior (constant passage width) when plotted as a function of the post diameter. In the case of monotonic trend, the width of interstitial spaces increases with the increasing chain occupation number, while, in the case of non-monotonic trend, the width of interstitial spaces decreases with the increasing chain occupation number. In comparison with linear topology, for circular topology, the stiffness affects more significantly the relative chain extension along the posts and less significantly the occupation number. The geometrical parameters of the post arrays are stored in the single-chain structure factors. The characteristic humps are recognized in the structure factor which ensue from the local increase in the density of segments in the circular chains presented in an interstitial volume or from the correlation of parallel chain fragments separated by a row of posts. Although the orientation correlations provide qualitative information about the chain topology and the character of confinement within a single interstitial volume, information about the array periodicity is missing.

## 1. Introduction

In living systems, the function of biomacromolecules is dictated by their conformation, which, in turn, is considerably modified in geometrically confined or crowded spaces. Understanding of the biochemical processes at the molecular level requires detailed knowledge of the conformations of involved biomacromolecules. Nowadays, the single-chain experiments realized in nano- and microfluidic devices [1,2] provide static and dynamic properties of a confined biopolymer such as a DNA and, thus, the theoretically derived results can be compared with their experimental counterparts. The structure of a flexible or semiflexible linear chain confined in simple geometries such as a slit [3,4,5,6,7,8,9], channel [10,11,12,13,14,15,16,17,18,19,20,21,22,23,24,25,26,27,28,29] or spherical cavity [30,31,32,33,34] has been well explored using extensive theoretical, simulation and experimental studies. The related issues also include the translocation of a chain through a pore between interconnected cavities [35,36,37,38,39,40] mimicking the translocation of biomacromolecules across biological membrane or transport mechanism of drug delivery, and the entropy-driven segregation of polymer chains in confining spaces relevant to bacterial chromosome replication and separation upon cell division. Although biomacromolecules of circular topology such as a bacterial DNA or mitochondrial DNA of eukaryotes are quite abundant in biological systems, much less attention is paid to circular polymers in the scientific community.

A special situation arises when a chain is confined in an array of collinearly organized posts. In such an array, the interstitial space among four neighboring posts might be envisioned as a quasi-channel and the passage between two closest posts might be locally considered as a quasi-slit. The resulting chain conformation is governed by the geometrical quasi-biaxial confinement coupled with the translocation of a chain through the passage aperture and it is readily controlled by the separation, size, and packing manner of the nanoposts. Sufficiently separated nanoposts do not prevent a chain from the lateral expansion, in the directions perpendicular to the axes of posts. Instead, the nanoposts function as obstacles hindering the lateral motion of a chain and imposing unique structural behavior of a confined chain.

The experimental application of an array of nanoposts in electrophoretic separation was first described in 1992 [41]. The effect of hexagonal arrangement of microlithographic nanoposts on the elongation of DNA molecules during electrophoresis was shown to be comparable with the effect of a gel matrix and these arrays were suggested as efficient substitutes for gel matrices in order to extend the application of electrophoresis. This pioneering study was followed by plenty of experimental studies that pointed out further conveniences of using nanopost arrays in the separation procedure of DNA molecules [42,43,44,45,46,47]. The advantages of the hexagonal array of nanoposts in the electrophoretic separation of DNA molecules were pointed out because of the fast separation of DNA molecules of all lengths with high resolution [45]. The range of applicability for given molecular lengths is readily tunable through adjusting the arrangement, size and separation of the pillars, as well as the applied electric field, and the pulse time. Intriguing generation of nanoposts has been reported in Ref. [48]. In this work, the array of pillars is reversibly formed from a suspension of superparamagnetic particles with the applied homogeneous magnetic field. The spacing between nanopillars is easily tunable from <1 to ~100 μm distances through variation of the paramagnetic particles. This experimental setup does not need any microlithographic manufacturing.

The effect of electric field on the collisions of a chain with a single post was studied using computer simulations [49,50,51,52,53] and microfabricated experimental devices [54] to comprehend the phenomena behind the electrophoresis in microlithographic arrays of nanoposts. The transferability of the results from a single-post model to an array of regularly and irregularly arranged nanoposts was confirmed only for an array of very sparsely distributed nanoposts [55]. However, these results provide valuable information on the conformational deformation of a DNA molecule exposed to an external electric field in the presence of a cylindrical obstacle [54,56].

In this study, molecular dynamics simulations have been performed for a single circular and linear chain placed in an array of infinitely long collinear posts with a square lattice cross-sectional projection. Inspired by Brownian dynamics simulations of a single linear rather flexible chain in a dense array of nanoposts [57], the influence of the chain topology and stiffness on the conformation of a circular chain inserted in a post array is addressed. In this work, substantially longer chains are considered in order to approach the length of a DNA studied at the experimental level. Although a study of a stiffer linear chain representing a special case of a histone-complexed DNA in a post array has been already published [58], the behavior of neither flexible nor semiflexible circular chain confined in the array of nanoposts has been reported yet. There are neither other spatial constraints besides the posts imposed on a chain nor any interactions of the chains with the electric field considered in the present computer simulations. This is in contrast with the microfabricated post arrays employed in the electrophoretical separations [44,47], where the height of the posts and the width of the post array are limited by the sides of a rectangular microchannel.

In this work, the effect of the chain stiffness on the structure of a circular chain confined in an array of infinitely long parallel nanoposts is studied using coarse-grained molecular dynamics simulations. The posts are relatively densely distributed making a square-lattice cross-sectional arrangement. The comparison with a linear chain is also provided. The occupation number, chain span along the post axes, radius of gyration, single-chain structure factor and the orientation correlations are evaluated as functions of the geometrical parameters characterizing the post arrays. In a post array, a salient behavior of a circular chain is expected because of its closed topology and enhanced excluded volume interactions stemming from the increased local density of monomers when compared with the local chain density of a linear chain. In fact, the larger relative extension of a circular chain in the biaxial confinement in comparison to that of a linear chain has been already reported and explained in terms of the enhanced excluded volume effects operating in a confined circular chain [59,60,61,62]. In this study, the enhanced excluded volume interactions in a circular chain confined in a post array are observed in the axial chain extension and occupation number as well. The structure factor stores the information on the chain topology and also on the geometry of post array while the orientation correlations reflect exclusively the topological features.

## 2. Method and Model

In the present study, the effect of the chain stiffness and circular chain topology on the conformation of a polymer chain in the presence of a square array of parallel nanoposts is investigated using coarse-grained molecular dynamics (MD) simulations. The discretized bead-spring worm-like chain (WLC) model has been adopted to represent the semiflexible chains of both the linear and circular topology. In this model, a chain is built up of effective monomers (beads) consecutively connected by effective fluctuating bonds (springs). The same model without assuming the energy penalty due to deformation of the effective angles has been employed also for the representation of flexible chains. Since each bead is comprised of several atoms, the atomistic details are neglected in this model. Because the beads bear no charge, there are no components of electrostatic interactions in the total potential energy. The total potential energy of the system contains contributions due to the bond stretching, the bending of two consecutive effective bonds, and the non-bonded monomer–monomer and monomer–post pair interactions. 

**Figure 1:**
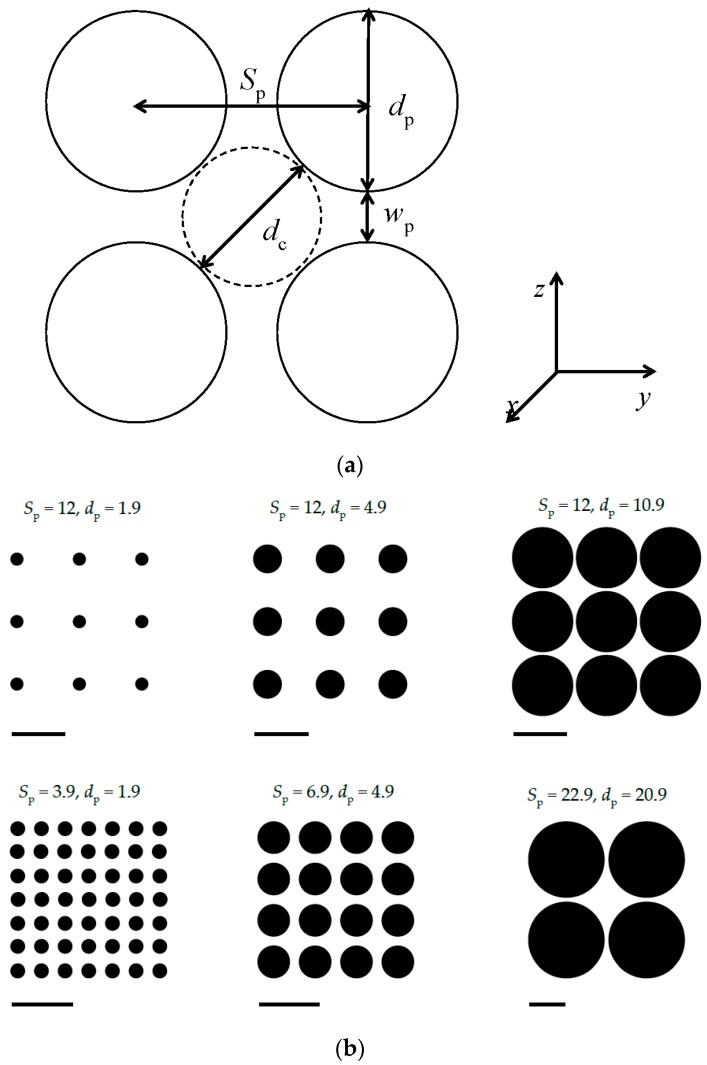
Cross-sectional view through four adjacent nanoposts defining the parameters of the nanopost square-lattice arrangement. *S*_p_ is the closest separation between the centers of two adjacent nanoposts, *d*_p_ is the diameter of nanoposts, *w*_p_ = *S*_p_ − *d*_p_ is the closest distance between the walls of two adjacent nanoposts, i.e., the passage width, and *d*_c_ = √2*S*_p_ − *d*_p_ is the diameter of a quasi-channel which is a gauge of the size of the interstitial space embraced by four neighboring nanoposts (**a**). Selected geometries of the post arrays with the constant post separation (upper row) and with the constant passage width (lower row). The lengths of the bars are 10σ (**b**).

The non-bonded interactions between effective monomers were represented by the entirely repulsive Weeks–Chandler–Anderson (WCA) potential

(1) $$U_{\mathrm{WCA}}\left(r_{ij}\right)=4\varepsilon \left[{\left(\frac{\sigma }{r_{ij}}\right)}^{12}-{\left(\frac{\sigma }{r_{ij}}\right)}^{6}\right]+\varepsilon$$

where the Lennard–Jones parameters ε = 1 and σ = 1 defined the interaction energy between the beads and the bead diameter, respectively, and *r_ij_* was the distance between bead *i* and *j*. Throughout this study, the Lennard–Jones parameters stand for the units of the energy and distance. To remove the attractive part of this potential, the condition $U_{\mathrm{WCA}}\left(r_{ij}\right)=0$ for $r_{ij}\geq 2^{1/6}\sigma$ is imposed on Equation (1). The interactions between beads and posts were treated by the same kind of repulsive interaction potential in the form

(2) $$U_{\mathrm{WCA}}\left(r\right)=4\varepsilon \left[{\left(\frac{\sigma }{r-D_{p}/2}\right)}^{12}-{\left(\frac{\sigma }{r-D_{p}/2}\right)}^{6}\right]+\varepsilon$$

where *r* is the distance between a bead and a post and *D*_p_ is the diameter of posts. As, in this study, the spacing between posts *S*_p_ used in these simulations is always larger than the interaction cut-off distance $r_{c}=2^{1/6}\sigma$, a bead could potentially interact with maximum of the four nearest posts (Figure 1).

The effective bonds were controlled with the finitely extensible nonlinear elastic (FENE) potential

(3) $$U_{\mathrm{FENE}}\left(l_{ij}\right)=-\frac{\kappa }{2}R_{o}^{2}\ln\left[1-{\left(\frac{l_{ij}}{R_{o}}\right)}^{2}\right]$$

where $l_{ij}$ is the effective bond length, $\kappa =30{\varepsilon \sigma }^{-2}$ is the spring constant and $R_{o}=1.5\sigma$ is the maximal alowable bond length. In addition to the FENE potential, two bonded beads interact also through the WCA potential (Equation (1)). The combination of these potentials yields the size of a bead *w* ≈ 0.9σ and the effective bond length *l* ≈ 0.97σ. Since *w* ≈ *l* this model belongs to the group of the touching bead models of polymer chains. The effective diameter of a post is thus enlarged to *d*_p_ = *D*_p_ + *w*. In this paper, *w* = *l* is considered in the expressions of investigated quantities and the respective plots.

The bending potential originated from the chain stiffness was derived from the elastic energy for a WLC in a continuum limit

(4) $$U_{\mathrm{WLC}}=\frac{1}{2}B\int_{0}^{L}ds{\left(\frac{du\left(s\right)}{ds}\right)}^{2}$$

where *s* is the arc length, ***u***(*s*) is the corresponding tangent unit vector, and *B* is the elastic constant related to the chain persistence length *P* = *B*/*k*_B_*T*. The discretized version of this WLC energy $\left(B/2l\right)\overset{N-1}{\underset{i=1}{\sum }}{\left(u_{i+1}-u_{i}\right)}^{2}\;$=$\;\left(B/l\right)\overset{N-1}{\underset{i=1}{\sum }}\left(1-u_{i}u_{i+1}\right)$ with *l* and ***u****_i_* being the bond length and the unit vector of *i*-th bond, respectively [63], leads to the commonly used form of the bending potential energy

(5) $$U_{b}\left(\theta \right)=\frac{B}{l}\left(1+\cos\theta \right)$$

where θ is the valence angle between two consecutive bonds in a chain (i.e., the complementary angle of $u_{i}u_{i+1}$). The dimensionless stiffness parameter, *b* = *B*/*lk*_B_*T*, was set to 20 for semiflexible chains.

The actual persistence length, *P*, for a free unconfined linear chain was determined from the bond orientation correlation function along the chain contour on a short length scale of the order of the persistence length using $\langle {\cos\theta }_{ij}\rangle =\exp\left[-\frac{l\left|i-j\right|}{P}\right]$, indices *i* and *j* stand for bond [28,64]. Value *P* = 19.724 obtained in this way for a free linear semiflexible chain agreed well with the expected value P=b〈l〉=20×0.97=19.4. In these simulations, the stiffness parameter *b* = 20 of the semiflexible chains corresponds to the value of the persistence length for a DNA molecule under high ionic strength conditions when *w* ≈ 2.5 nm; *P*/*w* ≈ 50 nm/2.5 nm = 20 since the real bending stiffness of a DNA molecule is approximately *B* = 189 pNnm^2^ the accepted persistence length is ~50 nm. This chain representation has been already successfully used for the MD simulations of a single-chain of both the linear and circular topology confined in a rectangular channel of various aspect ratios ranging from a slit-like to a rectangular channel [62].

Both the circular and linear chain comprised 1000 beads, which were connected altogether with 1000 and 999 effective bonds, respectively. For semiflexible chains, this contour length consists of L/P≅50 persistence lengths or ~25 Kuhn segments and corresponds to ~7.4 kb. For a free semiflexible linear chain, this number of persistence lengths has been shown to yield structural properties between the Gaussian and excluded volume statistics [65]. For comparison, the lengths of most frequently encountered DNA molecules in experimental measurements at physiological ionic strength contain ~300 (λ-DNA, 48.5 kbp) and ~1000 (T4-DNA, 165.6 kbp) persistence lengths.

**Figure 2:**
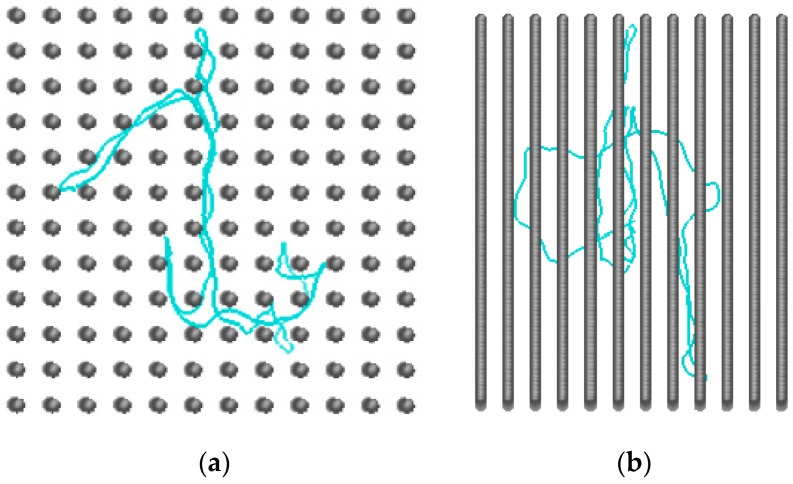
Snapshot of a semiflexible circular chain in an array of nanoposts with *d*_p_ = 4.9 and *S*_p_ = 12: top view (**a**); and side view (**b**).

The array of collinear posts was organized in the pattern of a square lattice. The post axes were aligned with the *x* coordinate. The cross-sectional view of the *yz* plane is outlined in Figure 1a. The spatial constraints were tuned in two ways: (1) the spacing between posts was kept constant at the value *S*_p_ = 12 while the post diameter *d*_p_ spanned the interval 1.9–11.9; and (2) the passage width was set to the constant value *w*_p_ = 2 while the *S*_p_ and *d*_p_ covered the ranges 3.9–62.9 and 1.9–60.9, respectively. Some representative examples of both series of geometry variation are illustrated in Figure 1b. One should notice, that when the post separation is kept constant there are limitations in the values of the post diameter, *d*_p_ ≤ *S*_p_, and thus the interstitial space, *d*_c_ ≥ (√2 − 1)*S*_p_. The quantification of constraining strength is based on two parameters, *d*_c_ and *w*_p_. The geometrical parameters of the post arrays are collected in Table 1 along with two quantities incorporating both parameters, *d*_c_ and *w*_p_, which are the volume fraction of the posts defined as $F=\pi d_{p}^{2}/4S_{p}^{2}$, and the ratio $d_{c}/w_{p}$. It should be stressed at this point that, in almost all geometries, the diameter of the quasi-channel *d*_c_ representing the size of the interstitial space and the width of the passage aperture *w*_p_ are smaller than the persistence length of a free semiflexible chain, *P* = 19.7. The only two exceptions are the post arrays with parameters *S*_p_ = 52.9, *d*_p_ = 50.9 and *S*_p_ = 62.9, *d*_p_ = 60.9 with *d*_c_ = 23.9 and 28.1, respectively. This arrangement means rather tight spacing of the posts when compared with the nanopost spacing common in experimental electrophoretical devices. The separation of adjacent nanoposts in microfabricated arrays is usually several multiples of the DNA persistence length [43,44,45,46,47,48] often reaching the micro scale. In these simulations, a polymer chain has a complete freedom in the direction parallel to the axes of posts. There are no restricting walls, neither in the parallel nor in the perpendicular directions with respect to the post axes. In principle, no periodic boundary conditions were necessary to be imposed in the simulated systems. A snapshot of the semiflexible circular chain confined in the array of nanoposts of diameter *d*_p_ = 4.9 spaced by *S*_p_ = 12 is shown in Figure 2.

The initial conformation of the linear chain was assumed with the completely straightened backbone and the initial conformation of the circular chain was assumed as completely prolonged rectangular loop with the side lengths *L*/2 and *l*. Two initial orientations of these straightened conformations were considered; one orientation with the parallel alignment of the chain principal axis with the post axes, and the second orientation with the chain principal axis orientated perpendicularly to the post axes. After equilibration both initial orientations for both topologies provided virtually identical structural properties.

All the MD simulations were performed using the DL Poly Classic package (STFC Daresbury Laboratory, Daresbury, UK) [66]. The systems were simulated in an NVT (constant number of particles, volume and temperature) ensemble where the temperature was kept constant at *T* = ε/*k*_B_ applying the Nosé–Hoover thermostat with a relaxation time of 0.1τ. The time unit was expressed in the simulation units as τ = σ (*m*_o_/ε)^1/2^, with *m*_o_ = 1 being the unit of mass. The time step was set to 0.005τ. The leap-frog algorithm was implemented for the numerical integration of the chain trajectory. After a thermal pre-equilibration lasting 10^5^τ the system was equilibrated at *T* = 1ε/*k*_B_ for 5 × 10^7^τ. During this equilibration, the satisfactory oscillations of the potential energy and radius of gyration were achieved. The equilibration was followed by the production phase which lasted for 2 × 10^8^τ. For the analyses of the structural quantities, 2 × 10^4^ conformations were considered, the frames were collected every 10,000th step. For each system, the resulting properties were obtained as the average over the all collected frames from three independent simulations. The size of the chains modified with the post array was characterized by the mean span of the chain along the post axes defined as

(6) $$R_{s}\equiv \langle \max\left(x_{i}\right)-\min(x_{i})\rangle$$

where i∈[1,N], *N* is the total number of monomers in a chain and *x*_i_ is the *x* coordinate of the *i*-th monomer. This is an experimentally observable quantity and, in contrast to the end-to-end distance, it is defined also for a chain with a closed topology. As the channel-induced stretching of a linear chain increases, the mean span approaches the end-to-end distance [19]. In order to study the asymmetry of the chain dimensions induced by the confinements, the mean-square radius of gyration $R_{g}=\langle R_{g}^{2}\rangle^{1/2}$ was also calculated and decomposed into the longitudinal, $R_{\mathrm{gx}}=\langle R_{\mathrm{gx}}^{2}\rangle^{1/2}$, and lateral, $R_{\mathrm{gyz}}=\langle \left(R_{\mathrm{gy}}^{2}+R_{\mathrm{gz}}^{2}\right)/2\rangle^{1/2}$, components. Partitioning of the chain monomers among the interstitial volumes in the array of nanoposts was quantified by the occupation number which counted the number of interstitial spaces occupied by the monomers.

With the purpose of the calculation of occupation number, the cross-sectional area of the post array was compartmented into space-filling squares with their vertices coinciding with the post centers. These squares delimited the individual interstitial spaces. Thus, even a monomer occurring in a passage between two posts was counted as occupying a respective interstitial volume. The internal organization of monomers within a circular chain was studied using the single-chain structure factor and the orientation correlation function. The free circular and linear chain composed of 1000 effective beads were considered as the references.

## 3. Results and Discussion

### 3.1. Occupation Number

**Figure 3:**
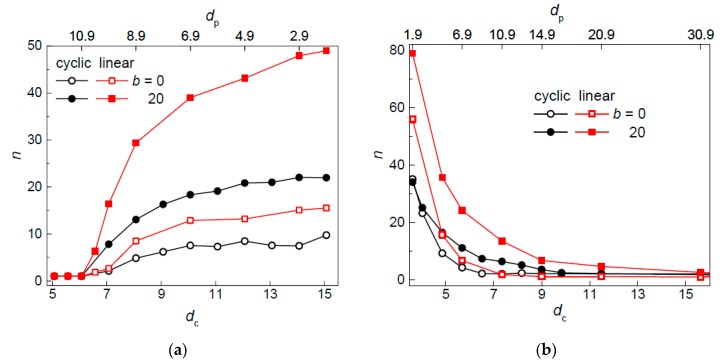
Occupation number of a flexible (*b* = 0) and semiflexible (*b* = 20) circular and linear chain in an array of posts of varying diameter *d*_p_ separated by *S*_p_ = 12 (**a**) and with passage width *w*_p_ = 2 (**b**) as a function of the size of the interstitial space.

As has already been mentioned above, the constraints are modified through alteration of the post diameter *d*_p_ while keeping the post separation at the constant value *S*_p_ = 12 or keeping the passage width at the constant value *w*_p_ = 2. The corresponding changes in the volume fraction of nanoposts cover almost two orders of magnitude (Table 1). The structural behavior of a chain is determined by the segregation of chain monomers into the interstitial volumes of nanopost arrays. The occupation number of the circular chain is compared with the occupation number of the analogous linear chain in Figure 3a,b where it is plotted as a function of the size of the interstitial space *d*_c_ linked with the respective post diameter *d*_p_ (upper abscissa) for the arrays with fixed *S*_p_ and *w*_p_, respectively. The occupation number of the analogous linear chains is also included for the comparison.

Apparently, as the width of the interstitial space increases with the reduction of the post diameter in Figure 3a, a chain is spread over more interstitial spaces. The trend is, however, opposite in Figure 3b, where the chain occupation number drops down with the increasing size of the interstitial space which in turn is now induced by the increase in the post diameter. The different trends in the occupation number with respect to *d*_c_ parameter can be interpreted in terms of the ratio *d*_c_/*w*_p_. As can be seen in Table 1, this ratio increases with increasing post diameter in both kinds of geometry variation. It follows that the trend in the occupation number is dictated by the *d*_c_/*w*_p_ ratio or alternatively the volume fraction of posts (Table 1). The single interstitial volume occupancy becomes more favorable at higher values of the *d*_c_/*w*_p_ ratio. The semi-quantitative explanation resorts to the approximation of the interstitial space and the passage aperture to channel and slit geometries, respectively. It has been established that the free energy of a linear chain confined in a channel of diameter *D* is twice the free energy of a chain confined in a slit of height *D* [67,68]. For a flexible chain, the de Gennes regime determines the conformation of a chain in a channel as well as in a slit. Assuming partitioning of a chain between an ideal channel and an ideal slit, the free energy scales as *d*_c_^−5/3^ and *w*_p_^−5/3^, respectively. The penetration of a chain from an interstitial space into a passage aperture happens when the free energy barrier vanishes which implies that 2*d*_c_^−5/3^ = *w*_p_^−5/3^. This predicts that the chain penetration into more interstitial spaces initiates below *d*_c_/*w*_p_ ≅ 1.52. One can see in Figure 3 and Table 2 that the corresponding values obtained from the simulation results are uniformly higher. The situation for semiflexible chains is more complicated since, in addition to the de Gennes regime [69,70], the Odijk regime [10] might occur in very narrow spaces. These larger values of *d*_c_/*w*_p_ than the predicted ones might originate from less severe confinement restrictions imposed on the chain fragments located in the passage aperture than the restriction imposed by a corresponding slit. Interesting is the comparison of the ratio *d*_c_/*w*_p_ under which the chain penetrates into more interstitial spaces between the flexible circular and linear chain in the post array of the fixed passage width, i.e., 14.0 vs. 9.9 (Table 2). This trend is opposite to what is expected, considering the larger size of the linear chain when compared with the circular analogs. The larger value for the circular chain indicates increased excluded volume interaction present in this chain and thus it promotes the circular chain to expand also laterally into more interstitial volumes already at larger *d*_c_/*w*_p_ or larger volume fraction of posts *F*. The chain stiffness diminishes the excluded volume interactions between chain segments and consequently the difference in the value *d*_c_/*w*_p_ ≈ 14 of the transition from single to multiple occupancy is more or less the same for both chain topologies. The linear chains segregate into more interstitial spaces when compared with the analogous circular chains. Nevertheless, considering the effective size of a bead to be *w* ≈ 0.9, exclusively single-interstitial space occupancy is expected below the passage width of this value. This applies to the geometries of the post array with the constant post separation *S*_p_ = 12 and *d*_p_ > 11.1.

The occupancy of the flexible linear chain is almost double the occupancy of the flexible circular chain and even more than double when the semiflexible linear chain is compared with the semiflexible circular chain. For both topologies, the stiffer chains penetrate into more interstitial volumes for the same geometries of nanopost array. The effect of the chain stiffness on the chain occupancy is more significant for the linear chains. What might seem to be intriguing is that the stiffness of a chain promotes chain penetration into more interstitial spaces. During the lateral expansion (with respect to the post axes) of a chain into more interstitial spaces, chain fragments have to be translocated through the passage apertures which essentially act as local uniaxial confining spaces. The free energy of a semiflexible linear chain in a slit-like confinement is supposed to scale as *P*^−α^. Although different values of α have been reported, namely α = 1/3 [9,10] or α = 1/2 [71], a fragment of the stiffer chain experiences less energy penalty when being translocated through a passage aperture.

### 3.2. Chain Extension and Radius of Gyration

**Figure 4:**
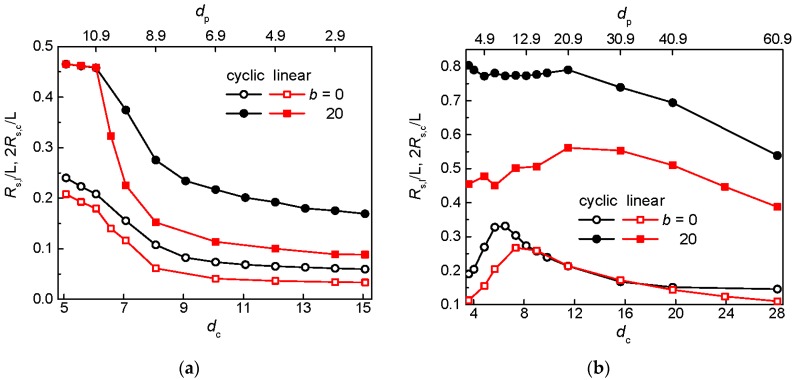
Relative extension along the posts for a flexible (*b* = 0) and semiflexible (*b* = 20) circular and linear chain in an array of posts of varying diameter *d*_p_ separated by *S*_p_ = 12 (**a**) and with passage width *w*_p_ = 2 (**b**) as a function of the size of the interstitial space *d*_c_.

The extension of a circular and linear chain along the post axes reduced by the maximal chain extension in different post arrays is presented for the both chain topologies at constant *S*_p_ and *w*_p_ in Figure 4a,b, respectively. As the size of the interstitial space increases the relative span of the chains monotonically decreases in Figure 4a consistently with the enhanced chain occupancy shown in Figure 3a. The effect on the axial chain extension due to the enhanced chain occupancy is the same as due to the increased interstitial space. As the occupation number increases a chain expands more in lateral directions and thus contracts in the longitudinal direction. At the same time, the larger size of the interstitial space promotes the relaxation of chain fragments in the lateral directions with concomitant axial contraction. Thus, the axial chain extension decreases with decreasing ratio *d*_c_/*w*_p_. The relative chain expansions of a flexible circular and linear chain display parallel behavior over all range of post diameters while there is a collapse of the curves for a semiflexible circular and linear chain in the arrays of wide posts with *d*_p_ > 1.

The variation of the relative chain extension is non-monotonic when the geometry of the nanopost array is modified at constant passage width as can be deduced from Figure 4b. Similar to previous simulations [57], there is a maximum in the plots for both topologies of flexible chains. In contrast with the variation of the post geometry at constant post separation, the decrease of the occupation number is accompanied with the increase of the size of the interstitial space at constant passage width. These effects influence the axial chain extension in the opposite way. The former effect promotes the axial chain extension while the latter effect enables the lateral chain relaxation. At small occupation numbers (large *d*_p_), the effect of the quasi-biaxial confinement in an individual interstitial space dominates and the relative span of the chain increases as the size of the interstitial space decreases. After the maximum, the span drops down with further increase of the occupation number. Now, the effect of the lateral chain expansion into more interstitial volumes prevails over the quasi-channel induced stretching of the chain. The chain closure restricts the circular chain to smaller space (lower occupation number) and the maximum in the relative chain extension is achieved at smaller *d*_c_ values. The plot for the semiflexible linear chain exhibits behavior similar to the plot for the flexible chains, although it is flatter. Interesting is the plot for the semiflexible circular chain where the effect of a quasi-channel-induced longitudinal extension prevails again at large occupation numbers (small *d*_p_) due to the enhanced excluded volume effects present in a confined circular chain.

One can also see that the effect of the chain stiffness on the relative chain extension is more striking for the circular topology which is in contrast with the occupation number where the stiffness plays more significant role for chains of the linear topology. The enhanced relative channel-induced chain extension observed for circular chains agrees with the earlier studies [59,60,61] and is attributed to the enhanced self-excluded interactions in a chain of the circular topology under confinement due to the increased local density of monomers.

**Figure 5:**
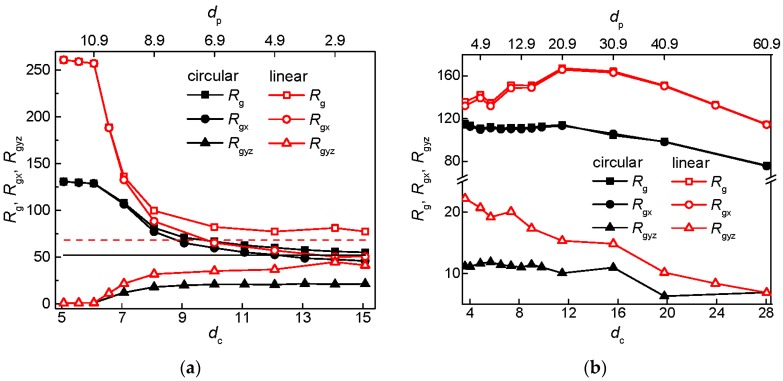
Radius of gyration and its longitudinal *R*_gx_ and perpendicular *R*_gyz_ components of a semiflexible circular and linear chain in an array of posts of varying diameter *d*_p_ separated by *S*_p_ = 12 (**a**) and with passage width *w*_p_ = 2 (**b**) as a function of the size of the interstitial space. The solid and dashed horizontal lines in (**a**) denote the value of the radius of gyration of a corresponding free circular and linear chain, respectively.

The asymmetry of the chain dimensions induced by the post array is well documented for the semiflexible circular and linear chain in Figure 5a,b, for the series with constant *S*_p_ and *w*_p_, respectively, which show the behavior of the radius of gyration and its longitudinal *R*_gx_ and perpendicular *R*_gyz_ components as functions of the size of the interstitial space or post diameter. In the array of broad posts, the overall radius of gyration is dominated by the longitudinal contribution in Figure 5a. This dominance extends practically over the all range of investigated *d*_c_ values in Figure 5b. The variation of *R*_g_ and *R*_gx_ qualitatively copies the variation of *R*_s_ in both plots of Figure 5. The perpendicular component *R*_gyz_ is practically governed by the size of the interstitial space as can be seen from the collapse of the curve for a circular and linear chain at the single interstitial space occupancy when the post diameter is sufficiently large in Figure 5a,b. Upon decreasing the post diameter, the value of the longitudinal component tends to drop down while the value of the perpendicular component rises in Figure 5a. For the linear chain in an array of narrow posts, both components of the radius of gyration attain almost identical value ~*R*_g_/√3 as expected for a free chain. The radius of gyration also approaches the value calculated for a free linear chain. This tendency is significantly attenuated for the circular chain and is not so obvious in Figure 5b where the components of the radius of gyration seem to be far from the convergence to the free-chain values at *w*_p_ = 2 and *d*_p_ = 1.9. This is not surprising when taking into account that, at the constant post separation *S*_p_ = 12, the narrow posts in Figure 5a provide a much broader passage width than the constant value *w*_p_ = 2 in Figure 5b. For instance at the constant post separation, the passage width for the nanoposts with *d*_p_ = 1.9 is *w*_p_ = 10.1. For the circular chain, the minor increase in *R*_g_ and *R*_gx_ and concomitant decrease in *R*_gyz_ at small values of *d*_c_ in Figure 5b are in line with the minor increase in *R*_s_ presented in Figure 4b and discussed in the previous paragraph.

**Figure 6:**
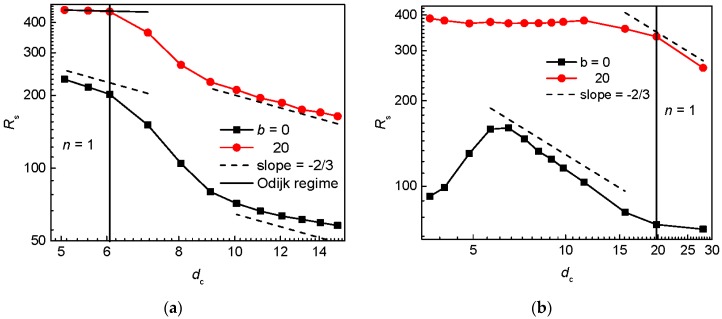
Extension along the posts for a flexible and semiflexible circular chain in an array of posts of varying diameter *d*_p_ separated by *S*_p_ = 12 (**a**) and with passage width *w*_p_ = 2 (**b**) as a function of the size of the interstitial space in a logarithmic scale. The solid vertical line demarcates the region with the dominance of single-occupancy for a semiflexible chain.

To validate the quasi-channel approximation of the interstitial spaces, the extension of the flexible and semiflexible circular chain is plotted in a logarithmic scale against the diameter of the quasi-channel in Figure 6a,b for the series with constant *S*_p_ and *w*_p_, respectively. The behavior of a chain confined in a channel is well understood and different regimes according to the channel diameter *D* and chain parameters are recognized. The strong confinement known as the Odijk regime [10] operates when the chain persistence length exceeds the channel diameter P≫D. Under this condition, the deflection length $\lambda \approx {\left(D^{2}P\right)}^{1/3}$ defined as a rod-like segment between two consecutive collisions with the channel wall becomes the statistical segment of a chain. The longitudinal chain extension is defined as

(7) $$R=L\left[1-A{\left(\frac{D}{P}\right)}^{2/3}\right]$$

where *A* = 0.1701 is a numerical constant estimated for a cylindrical channel [18]. In the moderate confinement regime with P≪D, a chain is viewed as a string of blobs. In the extended de Gennes regime [19,72,73], $P<D<P^{2}/w$ and the blobs are anisometric while in the classic de Gennes regime [69,70], $P^{2}/w<D$ and the blobs are isometric. Although the free energy of a chain is different, the chain extension is defined by the same relation in both blob regimes

(8) $$R\approx L{\left(\frac{wP}{D^{2}}\right)}^{\frac{1}{3}}$$

The approximation with the channel-like confinement might be expected for geometries of post arrays in which the single-interstitial volume occupancy is preferred. In the case of the constant post separation, this applies for small *d*_c_ values (large post diameters). In Figure 6a, the approximation of the deflection Odijk regime fits really well for the semiflexible circular chain, if the half of the contour length *L* is assumed in Equation (7). The slope −2/3 characteristic for the moderate de Gennes regime, Equation (8), satisfactorily represents the behavior of the flexible circular chain at small values of *d*_c_. Interestingly, for the semiflexible chain, the de Gennes regime manifests itself for larger *d*_c_ values (smaller post diameters) at which the multi-interstitial volume occupancy occurs. At these *d*_c_ values, the occupation number is almost constant and one might consider this evolution of the chain expansion with the size of the interstitial space as the evolution of more chain fragments each occupying single interstitial volume. Since the de Gennes regime requires channel diameters larger than the chain persistence length, it seems that the confinement of a single interstitial space is more relaxed than the confinement in a channel with the diameter equal to *d*_c_.

There is an indication of the de Gennes regime if the passage width is held constant in Figure 6b. It is shifted to larger values of *d*_c_ for the semiflexible circular chain when compared with the flexible circular chain. Since the de Gennes regime is expected when a chain is restricted to only one interstitial space and this situation sets in at larger *d*_c_ values for the semiflexible chain such a shift is expected. Similarly to Figure 5a, there is a deviation from the slope −2/3 for large values *d*_c_. The existence of the Odijk regime is ruled out by large size of interstitial space needed in order to achieve a single-chain occupancy.

### 3.3. Structure Factor

The important information on the statistical organization of chain segments is stored in the single-chain structure factor. This quantity provides insight into the hierarchy of the chain organization on different length scales when plotted as a function of the wavevector *q* = 2π/Ω. Here, Ω represents the length scale ranging from the monomer size up to about the chain size. The structure factor is defined as follows

(9) $$S\left(q\right)=\frac{1}{N^{2}}\langle \overset{N}{\underset{i=1}{\sum }}\overset{N}{\underset{j=1}{\sum }}\sin\left(qr_{ij}\right)/qr_{ij}\rangle$$

where *r_ij_* is the distance between segments *i* and *j*. At small values of the wavevectors known as the Guinier regime, when *q* < 2π/*R_s_*, the structure factor displays a saturation. This regime is not important for presented analyses and its appearance is shifted to lower *q* values for more extended chains. The region in structure factor relevant for the analysis of the chain organization is at larger values of the wavevectors where *S*(*q*) ~ *q*^−1/*ν*^ (*ν* is the Flory scaling exponent). According to the channel size and geometry as well as the chain total contour length, persistence length and excluded volume interactions, i.e., the width of the chain backbone, a rich scale of slopes in the logarithmic plot of *S*(*q*) might appear [60,62,74,75].

The slopes expected in the structure factor for a chain confined in a symmetric channel, which serves as a model for the geometry of the interstitial space, depend on the conformational regime [62]. Generally, the persistence length of a chain gives rise to the slope −1 for *q* > 2π/*P*. In the blob regime, under moderate confinement effects, the statistics within a blob determines the slope value which depends on the regime. In the classic de Gennes regime, this slope is −5/3 and is delimitated by 2π/*P* > *q* > 2π/*D* for the channel diameters *D* > *P*^2^/*w*. In the extended de Gennes regime, the slope −2 is expected in the interval 2π/*P* > *q* > 2π/(*D*^2^*P*^2^/*w*)^1/3^ for the channel diameters *P*^2^/*w* > *D* > *P*. The slope −2 might, however, also point to the pseudoideal regime observed for shorter biaxially confined chains. At *q* < 2π/*D* (classic de Gennes regime) or at *q* < 2π/(*D*^2^*P*^2^/*w*)^1/3^ (extended de Gennes regime), the slope −1 signifies the linear arrangement of blobs under moderate confinement of a symmetrical channel. In the Odijk regime, the structure factor is governed by the slope −1 over all wavevectors *q* > 2π/*R*_s_. The appearance of all slopes in one plot of *S*(*q*) function requires a sufficiently long chain with a sufficient number of the persistence lengths confined in a channel which is broad enough for the blobs to be developed.

**Figure 7:**
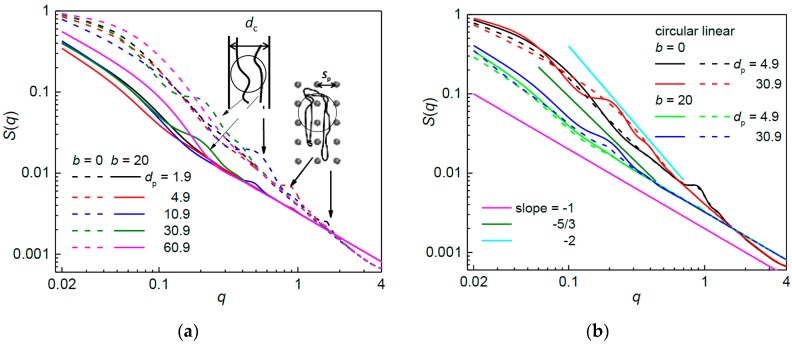
Structure factor for: a flexible and semiflexible circular chain (**a**); and for a flexible and semiflexible circular and linear chain (**b**), confined in an array of nanoposts of fixed passage width *w*_p_ = 2 and various post diameters *d*_p_. In (**a**), the insets represent the preferred chain conformation associated with the humps (on the left: due to the local increase in density, on the right: due to the parallel fragments in adjacent volumes such as U-turns); and, in (**b**), the solid lines represent the characteristic slopes that might be expected for a biaxially confined chain.

The best agreement between the conformation of a chain situated in an array of posts and in a channel comes about at a single occupancy, i.e., at large post diameters (Figure 3a,b). The salient feature in the structure factor of a biaxially confined circular chain is a hump which is associated with the local increase in density of chain segments due to the parallel alignment of chain strands forced by the channel geometry [62] as sketched in the left inset of Figure 7a. It has also been shown that the onset of this hump appears at 2π/*D* and thus it well correlates with the channel cross-sectional size *D*. The humps are evidently present also in the structure factor of the flexible circular chain confined in an array of nanoposts as shown in Figure 7a where the passage width is kept constant. However, the humps arise also in the arrays where the nanopost diameters are quite small and allow multi-interstitial space occupancy. Positions of these humps correlate with ~2π/*S*_p_ rather than with 2π/*d*_p_ and are present also in the structure factor for the linear analogs (Figure 7b). Therefore, the presence of these humps points to a parallel organization of two chain fragments located in adjacent interstitial volumes. An example of such a chain conformation imposed by the posts arrangement is a U-turn shown in the right inset of Figure 7a. As regards the fragment separation, two chain fragments within a chain conformation might correlate also over two or more rows of the posts. The chain conformations containing such fragments could be deduced from the presence of more humps at *q* ≈ 2π/*nS*_p_ where *n* is the number of post rows between two parallel arms. The existence of these fragments is, however, not detected in Figure 7a.

As the occupation number decreases with increasing post diameter, the intensity of the hump increases and, for *d*_p_ ≥ 6.9, *S*_p_ ≥ 8.9, the onset of the hump becomes better correlated with ~2π/*d*_c_. Moreover, this hump disappears for the flexible linear chain. From these indications follows that the appearance of substructures with parallel chain strands found in neighboring interstitial volumes is reduced when the chain is located in a smaller number of broader interstitial volumes and, instead, the structure factor reflects a local increase in density of a chain caused by a quasi-channel like geometry of the interstitial space. For instance, the hump for the flexible circular chain confined in the array of posts with *d*_p_ = 30.9 and *w*_p_ = 2 arises at *q* ≅ 0.32 (this value belongs to the region associated with the blobs as discussed below) corresponding to 2π/*q* ≅ 31.4; this value is close to the diameter of the interstitial space. Such a hump is missing for the flexible linear chain in the same post array (Table 1). In the array of broad nanoposts with *d*_p_ = 60.9 (single-interstitial space occupancy, not shown), the onset of the hump is most likely overlapped with the onset of the Guinier regime which sets in at relatively larger values of *q* due to the smaller chain dimensions in this array.

In the arrays of small post diameters *d*_p_ ≤ 4.9 and *S*_p_ ≤ 6.9, the formation of humps, arising from the U-shape and other substructures with correlating parallel chain fragments, in the structure factor of the semiflexible chains of both topologies is suppressed. Although the stiffness of a chain promotes its expansion into more interstitial volumes, it prevents the chain from formation of the U-shape conformations since they become energetically less favorable, especially, at smaller post separations, i.e., at smaller post diameters. The humps resulting from the local increase in density of the semiflexible circular chain in the arrays of *d*_p_ > 4.9 chain are present in the structure factor but, in comparison to the flexible circular chain, are shifted slightly towards lower *q* values. This is expected, since the greater stiffness is responsible for larger lateral expansion of a chain within a single interstitial volume.

The structure factors of the circular and linear chains in the array of constant passage width are compared in Figure 7b. The structure factors of the flexible linear chain also exhibit the hump characteristic for U-shape and other substructures with correlating parallel chain fragments substructures observed in the arrays of small post diameters. The slope −1 well represents the persistence length scale for the semiflexible chains of both topologies. The statistics within individual blobs in the interstitial volumes demonstrates itself only for the flexible chains and, as one can see for the post array with *d*_p_ = 30.9, the slope in this region is −2. This means that the statistics of a chain within the corresponding length scale of an individual interstitial space is ideal in this post array. This might correspond to the extended de Gennes regime with the blobs of an anisometric ellipsoid shape [19,72,73] or to the pseudoideal regime [16,19,76]. Interestingly, the structure factor of the semiflexible linear chain exhibits a hump in the post arrays of *d*_p_ = 20.9 and 30.9. Since no such humps occur for the flexible linear chain, these humps most likely follow from the hairpin structures observed also for semiflexible chains in channels of certain diameters [17].

**Figure 8:**
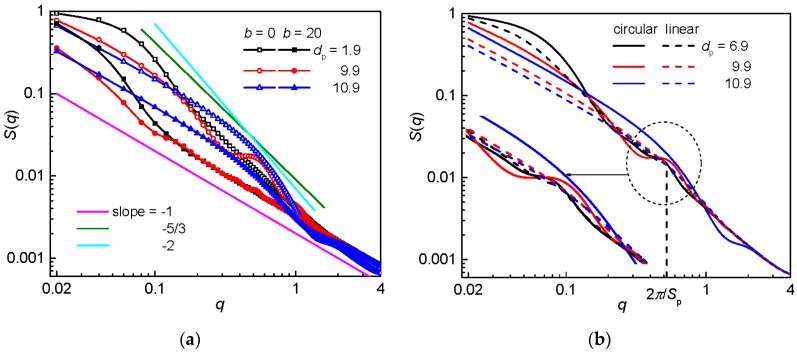
Structure factor for: a flexible and semiflexible circular chain (**a**); and for a flexible circular and linear chain (**b**) confined in an array of nanoposts of fixed post separation *S*_p_ = 12 and various post diameters *d*_p_. In (**a**), the solid lines represent the characteristic slopes that might be expected for a biaxially confined chain; and, in (**b**), the inset zooms in the region of humps with the vertical dashed line indicating its predicted onset.

The structure factors for a flexible and semiflexible circular chain confined in the post arrays of constant post separation are compared in Figure 8a. The hump characterizing the local increase in chain density in a quasi-channel due to the circular topology is formed only at large post diameter, *d*_p_ = 10.9, when the single-occupancy dominates. The onset of this hump at *q* ≈ 1 well correlates with the corresponding *d*_c_ in Table 1. In these nanopost arrays, the interstitial space is narrow, *d*_c_ = 7.1, and a chain is considerably extended in the axial direction which gives rise to the slope −1 also at smaller values of *q*. The hump in *S*(*q*) persists also for the circular flexible chain in the arrays of *d*_p_ = 9.9. For this variation of geometry, the values of *d*_c_ (Table 1) are smaller in comparison with *S*_p_ = 12 and one can recognize the origin of the onset of the hump by considering its position. Using this presumption along with the absence of an analogous hump in the structure factor for the flexible linear chain in Figure 8b leads to the conclusion that the hump in the structure factor for the flexible circular chain rather follows from the channel-induced local increase in density than from the substructures with neighboring parallel chain fragments. A little sign of a hump, which is observed in the structure factor for the semiflexible circular chain in the same geometry of the post array, coincides with the hump in the structure factor of the analogous semiflexible linear chain. From this follows that this little hump points to the substructures with neighboring parallel fragments. This is in line with the enhanced occupation number for a semiflexible chain. As the post diameter further decreases at constant post separation till *d*_p_ = 3.9, the hump in the structure factor due to the neighboring parallel fragments is detectable for the flexible circular and linear chain and practically disappears for the semiflexible chains. At constant *S*_p_, the onset of the hump (dashed horizontal line in Figure 8b corresponds to the prediction) for different sizes of nanoposts preserves constant (not shown) which is another indication of its origin. When the posts are very narrow (*d*_p_ = 1.9) and distinct from each other (*S*_p_ = 12), the structure factors for the flexible and semiflexible chains of both topologies completely match the structure factors for the respective free analogs (not shown). Comparison of these structure factors with slope −5/3 in the region of moderate *q* values really reveals the statistics expected for a free chain with excluded volume interactions. One could argue that this slope might also indicate the organization within a blob formed by the interstitial space, however, this would require *d*_c_ > *P*^2^/*w* ≈ 418, which is much broader than the values considered in the simulations.

### 3.4. Orientation Correlations

The tangent–tangent orientation correlation function has been used for the characterization of the internal orientation of chain segments. This quantity distinguishes the circular topology from the linear topology of a free [77,78] or confined chain [60,62]. For a free discretized WLC chain, the orientation correlation function between two unit vectors ***u***_i_ and ***u***_j_ tangent to the chain contour at the positions of *i*-th and *j*-th segment is defined as $\langle u_{i}u_{j}\rangle =\langle {\cos\theta }_{\mathrm{ij}}\rangle =\exp\left[-\left|j-i\right|l_{\mathrm{ij}}/P\right]$. The average covers all the equilibrated chain conformations and all chain fragments separated by *n*_s_ = |*i* − *j*| bonds. The exponential decay applies only to small chain fragments of the order of the persistence length for a chain with the excluded volume [79]. The analytical theory relates the orientation correlations with the chain persistence length and the channel size only for a strongly confined semiflexible linear chain pertaining to the Odijk regime [74,78,80]. For a biaxially confined linear chain, the characteristic shape of the orientation correlations is expressed as a periodic function with damped amplitudes

(10) $$\langle u_{i}u_{i+n_{s}}\rangle =1-\frac{\lambda }{2P}\left[1+\sqrt{2}\exp\left[-n_{S}l/\lambda \right]\sin\left(\frac{n_{S}l}{\lambda }-\frac{\pi }{4}\right)\right]$$

The length of the amplitudes, πλ, correlates with the deflection length λ = *c*(*PD*^2^)^1/3^. The most significant amplitude is the first minimum, which reflects the first deflection of a chain from the channel wall. The applicability of this relation for the orientation correlations of a semiflexible linear chain confined in a single interstitial space is, however, questionable. The orientation correlations of the circular chain display inversion symmetry and, to the best of our knowledge, there is no analytical expression relating the chain and confinement parameters with the orientation correlations for a chain of circular topology.

**Figure 9:**
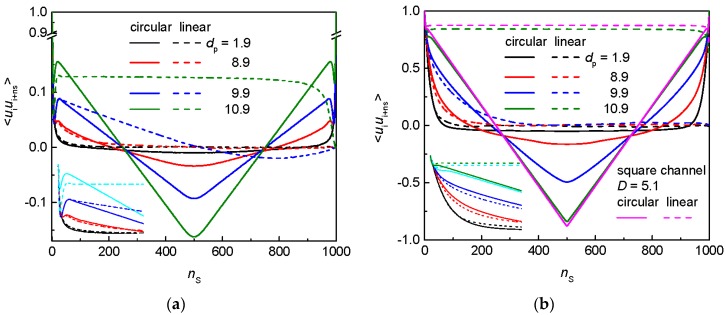
Orientation correlations for: a flexible chain (**a**); and a semiflexible chain (**b**) of circular and linear topology in an array of nanoposts of fixed post separation *S*_p_ = 12 and various post diameters *d*_p_. The insets zoom in the initial decay of the orientation correlations.

The orientation correlations for the flexible and semiflexible circular chains in the nanopost arrays of the constant post separation are compared with the orientation correlations of their linear analog in Figure 9a,b, respectively. In fact, the orientation correlations for the flexible circular chain in the post arrays of *d*_p_ < 8.9 are identical with those for a free flexible circular chain (not shown). This applies also to the flexible linear chain. Moreover, besides the large distance between the bonds, *n*_s_ → 1000, there is only a marginal difference between the orientation correlations in the central region for the circular and linear topology in the arrays with these post diameters. Nevertheless, as the chains are getting to be more restricted to a smaller number of the interstitial spaces, the distinction between the orientation correlations of the confined and free analogs as well as between the circular and linear topology becomes more marked. As the interstitial spaces are tightened due to the increasing post diameter, the initial decay of the orientation correlations passes into a maximum whose position decreases and whose amplitude increases with the decrease of the interstitial spaces. This maximum follows from the first chain collision with the post walls which explains the position order. After the maximum, one can observe a steadily declining trend toward the central minimum which is most profound and sharp for the narrowest interstitial space. These results are in consistency with the earlier findings in the simulated orientation correlations for chains of the both topologies in cylindrical confinement [60].

There is only a marginal difference in the orientation correlations for the semiflexible chain of the both topologies in the posts arrays with *d*_p_ < 7.9 and the free analogs (not shown). Akin to the flexible chains, the effect of the post on the chain geometry becomes significant in the arrays of wider posts (narrower interstitial spaces) when the topological differentiation also appears in the first half of the plots. Comparison of the orientation correlations between the both chain topologies in the array of broad posts (*d*_p_ = 10.9) with the orientation correlations of their analogs in a cylindrical channel of the diameter *D* = 5.1 ≈ *d*_c_ reveals a perfect agreement. The analytic relationship, Equation (10), also well represents the orientation correlations of the semiflexible linear chain in a single interstitial volume. The ensuing fitting parameters are *P* = 19.61 and *c* = 0.51.

**Figure 10:**
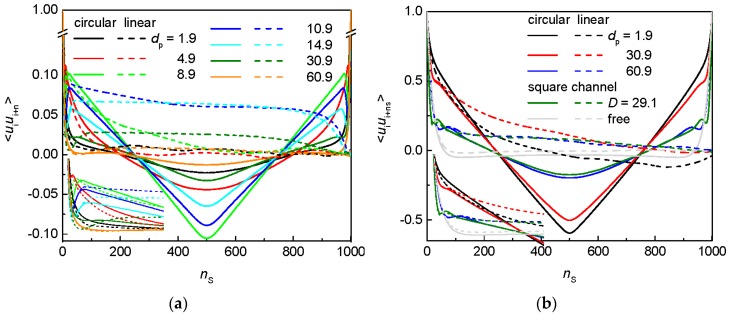
Orientation correlations for: a flexible chain (**a**); and a semiflexible chain (**b**) of circular and linear topology in an array of nanoposts of fixed passage width *w*_p_ = 2 and various post diameters *d*_p_. The insets zoom in the initial decay of the orientation correlations.

As one can see in Figure 10a, when the geometry of the post array is varied at the constant narrow passage width, the trend in the orientation correlations for the flexible circular and liner chain is irregular similarly to the non-monotonic trend observed for the chain span (Figure 4b). For the array of narrow posts with *d*_p_ = 1.9, the orientation correlations of the confined circular chain slightly differ from the orientation correlations of the free analog (not shown) since the passage width *w*_p_ = 2 is quite narrow. As the posts are widened at the constant passage width, the chain becomes more restricted to a smaller number of the interstitial volumes and the shape of the orientation correlations starts resembling more the shape for a circular chain confined in a channel. After the initial decay, the peak arises in the post arrays with *d*_p_ ≥ 4.9. The position of this peak is shifted to larger values, its amplitude is reduced and the central minimum is gradually deepened with further increase in the post diameter till *d*_p_ = 8.9. This peak is more significant for the circular chain since the terminal arcs of a circular chain also contribute to the increased values of the orientation correlations. Such a transformation of the orientation correlations reflects an increasing concentration of the chain segments in a single interstitial volume the size of which is concomitantly enlarging. Further broadening of the posts, however, alters the trend in the orientation correlations. Since this geometry variation means also broadening of the interstitial spaces the trend in the orientation correlations corresponds to the relaxation of a channel-like confinement, i.e., the position of the maximum is shifted to larger values and its amplitude but also the amplitude of the minimum are reduced. It should be mentioned at this point that the occupation number for these geometries of the post arrays does not exceeds two interstitial volumes. This trend continues till the chain orientation correlations being very close to those for a free chain are retrieved in the array of posts with *d*_p_ = 60.9. The alteration of the trend of the orientation correlations well coincides with the position of the maximum in the span shown in Figure 4b at which the priority of the two factors affecting the axial chain extension is altered. Worth noticing is the vanishing value of the orientation correlations at *n*_s_ ≈ *N*/4 and *n*_s_ ≈ 3*N*/4 for rings, also observed and explained in Ref. [62]. The orientation correlations for the flexible linear chain exhibit the same trend as those for the flexible circular chain. The most significant difference between the circular and linear topology in the initial parts of the orientation correlations is found for the arrays of *d*_p_ = 8.9 and 10.9 where the effect of the axial chain expansion in a single interstitial volume starts to prevail. This happens already at smaller post diameters in the case of the circular topology.

The orientation correlations for the semiflexible circular and linear chain, shown in Figure 10b, exhibit less sensitivity to the variation of the posts separation at the constant passage width than the orientation correlations for the flexible chains, in line with Figure 4b. However, there is a larger difference from the free analog for small *d*_p_ = 1.9 as well as large *d*_p_ = 60.9. The shape of the orientation correlations of the chain occupying only a single volume corresponds to the shape for a chain confined in a channel which is confirmed for the array of the post diameter *d*_p_ = 60.9 and *d*_c_ = 28.1 when compared with a circular chain in a square channel of *D* = 29.1. There is a good agreement for both topologies besides the position of the initial peak which indicates effectively larger diameter of a quasi-channel representation of the interstitial space. In fact, the maximum associated with the chain deflection is developed only for the largest investigated post diameters *d*_p_ = 40.9 and 60.9 where the chain is strictly located in a single interstitial space.

## 4. Conclusions

The effect of a constrained space within the array of parallel nanoposts organized in a square lattice on the structural properties of a circular chain has been investigated using the coarse-grained molecular dynamics simulations. To examine the stiffness effect, flexible and semiflexible circular chains have been studied and the effect of the closed chain topology has been addressed through the comparison with analogous linear chains. To the best of our knowledge, no such study has been reported thus far.

The geometry of the array of nanoposts has been modified in two ways: the post diameter, *d*_p_, has been varied at the constant post separation, *S*_p_, or the post diameter and the post separation have been varied to keep the passage width, *w*_p_, constant. At the constant post separation, an increase of the post diameter leads to a decrease of the diameter of a quasi-channel characterizing the size of the interstitial space, *d*_c_, while at the constant passage width, increasing of the post diameter is connected with an increase of the interstitial space. The number of interstitial volumes occupied by chain segments raises with decreasing ratio *d*_c_/*w*_p_. The transition from single-volume to multiple-volume occupancy has been roughly estimated using a channel approximation for the interstitial space and a slit approximation for the passage aperture and compared with the simulated data. Uniformly larger *d*_c_/*w*_p_ values than the values predicted for the chain transition from a single-volume to multiple-volume occupancy are obtained. The semiflexible chains of both topologies penetrate into more interstitial volumes more readily than the flexible analogs.

The evolution of the chain span is monotonically decreasing with the increasing post diameter for the post arrays with the constant post separation while a non-monotonic trend is found for the chain span when the post diameter increases at constant passage width. This difference in trends is explained in terms of two effects determining the resulting span; the lateral expansion/contraction of a chain connected with the increasing/decreasing occupation number, and the biaxial expansion/contraction of a chain in individual interstitial volumes connected with the decreasing/increasing *d*_c_. While in the case of the constant post separation both these effects act in the same way, in the case of the constant passage width these effects work oppositely. The non-monotonic trend in the latter case follows from the alteration of the preponderance between these two effects. The chain stiffness affects the relative chain extension more significantly if the chain topology is circular in contrast with the occupation number where the stiffness is more important for chains of the linear topology.

In fact, the variation of the chain span with the size of the interstitial spaces in the arrays of the constant post separation substantially resembles the variation of the chain span in a channel and the particular confinement regimes are identified for the flexible and semiflexible chains of the both topologies. In the arrays of constant passage width, the de Gennes regime is observed for the interval of *d*_c_ where the longitudinal chain extension is dominated by the biaxial restriction imposed by the interstitial volumes.

When plotted as a function of the wavevector, the structure factor reflects the geometrical parameters of the post arrays and discerns the closed and open chain topology. Two kinds of humps occur in the structure factors of the chains confined in the post arrays. The hump, typical for a biaxially confined circular chain ensuing from the local increase in density of chain fragments within a confining volume, arises in the structure factor at lower occupation numbers. The appearance of this hump is thus limited only to the arrays of wide posts. The intensity of this hump is always larger for the flexible circular chains, which agrees with their lower occupancy of the post arrays. The hump which reflects the post separation and results from the chain fragments separated by a row of the posts is developed for the flexible chains of both topologies, however, its presence in the structure factor of the semiflexible chains practically diminishes. Since bending about 180° in an array of posts is energetically less favorable for stiffer chains, there is only a small sign of the hump in the structure factor for the semiflexible chain in the arrays of larger post separations (*S*_p_ = 12).

The orientation correlations of the chains confined in the post arrays reflect the organization of chain segments within the individual interstitial volumes but the periodic arrangement of the posts is not detectable. As the chains are allowed to relax more in the post arrays, the differences between the chain topologies in the first half of the orientation correlations as well as between the confined and respective free analogs vanish. The orientation correlations for the chains occupying only a single volume well agree with those for a chain in a channel of diameter *D* ≈ *d*_c_.

All properties point out that the behavior of the flexible and semiflexible chains of both topologies approaches the behavior of free analogs in the arrays of narrow posts which are distant from each other (*d*_p_ = 1.9, *S*_p_ = 12).

## Figures and Tables

**Table 1:** Geometrical parameters of post arrays as defined in the text in the simulation length units; 1 unit ≈ 2.5 nm.

*S*_p_ = 12					*w*_p_ = 2				
*d*_p_	*w*_p_	*d*_c_	*F*	*d*_c_/*w*_p_	*d*_p_	*S*_p_	*d*_c_	*F*	*d*_c_/*w*_p_
1.9	10.1	15.071	0.020	1.492	1.9	3.9	3.615	0.186	1.808
2.9	9.1	14.071	0.046	1.546	2.9	4.9	4.030	0.275	2.015
3.9	8.1	13.071	0.083	1.614	4.9	6.9	4.858	0.396	2.429
4.9	7.1	12.071	0.131	1.700	6.9	8.9	5.687	0.472	2.843
5.9	6.1	11.071	0.190	1.815	8.9	10.9	6.515	0.524	3.257
6.9	5.1	10.071	0.260	1.975	10.9	12.9	7.343	0.561	3.672
7.9	4.1	9.071	0.340	2.212	12.9	14.9	8.172	0.589	4.086
8.9	3.1	8.071	0.432	2.603	14.9	16.9	9.000	0.611	4.500
9.9	2.1	7.071	0.535	3.367	16.9	18.9	9.829	0.628	4.914
10.9	1.1	6.071	0.648	5.519	20.9	22.9	11.485	0.654	5.743
11.4	0.6	5.571	0.709	9.284	30.9	32.9	15.628	0.693	7.814
11.9	0.1	5.071	0.772	50.706	40.9	42.9	19.770	0.714	9.885
					50.9	52.9	23.912	0.727	11.956
					60.9	62.9	28.054	0.736	14.027

**Table 2:** Values of the ratio *d*_c_/*w*_p_ under which the chains spread from single to multiple occupancy in the post arrays of the constant post separation *S*_p_ and of the constant passage width *w*_p_.

	**Circular**			
	*S*_p_ = 12		*w*_p_ = 2	
	*b* = 0	*b* = 20	*b* = 0	*b* = 20
*d*_c_/*w*_p_	5.5	5.5	14.0	14.0
	**Linear**			
	*S*_p_ = 12		*w*_p_ = 2	
	*b* = 0	*b* = 20	*b* = 0	*b* = 20
*d*_c_/*w*_p_	5.5	5.5	9.9	14.0
