# Correction: Benková, Z.; et al. Structural Behavior of a Semiflexible Polymer Chain in an Array of Nanoposts *Polymers* 2017, *9*, 313

**DOI:** 10.3390/polym9120665

**Published:** 2017-12-01

**Authors:** Zuzana Benková, Lucia Rišpanová, Peter Cifra

**Affiliations:** 1 Polymer Institute, Slovak Academy of Sciences, Dúbravská Cesta 9, 845 41 Bratislava, Slovakia; lucia.rispanova@savba.sk (L.R.); Peter.Cifra@savba.sk (P.C.); 2 LAQV@REQUIMTE, Department of Chemistry and Biochemistry, Faculty of Sciences, University of Porto, Rua do Campo Alegre 687, 4168-007 Porto, Portugal

There was a mistake in the original code evaluating the occupation number of polymers. That mistake resulted in overestimated values of occupation numbers as compared to values obtained after the mistake was corrected (compare original Figure 3 and new Figure 3). Figure 3a,b of the original manuscript should be replaced by Figure 3a,b of the correction. The reported overestimation of occupation numbers did not affect the qualitative discussion concerning the occupation number and the definition of the occupation number introduced in the main text of the manuscript. The general trends have remained the same as well. Table 2 in the original manuscript should be replaced by Table 2 of the correction. Moreover, the data in Table 2 pointing out values indicating when a chain penetrates into more interstitial spaces have changed only in two cases, which are corrected in the new version of Table 2.

One correction should be made also in the main text on p. 320, where 14.0 vs. 7.8 should be replaced with 14.0 vs. 9.9.

## Figures and Tables

**Figure 3:**
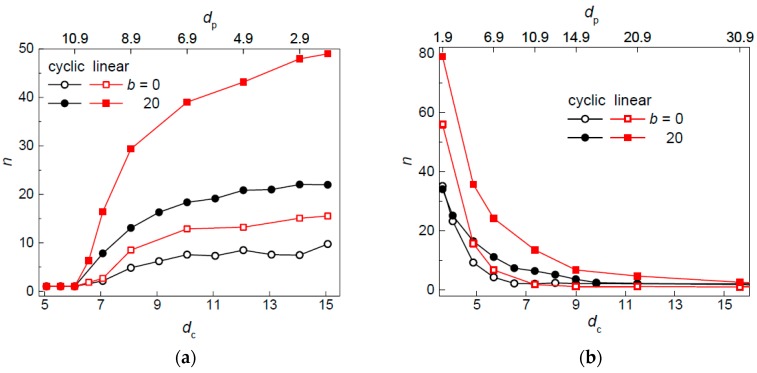
Occupation number of a flexible (*b* = 0) and semiflexible (*b* = 20) circular and linear chain in an array of posts of varying diameters *d*_p_ separated by *S*_p_ = 12 (**a**) and with passage width *w*_p_ = 2 (**b**) as a function of the size of the interstitial space. The trends in the upper abscissa are reversed when compared to 3a and 3b due to the different way of varying the geometry of the post array as defined in the main text of the manuscript.

**Table 2:** Values of the ratio *d*_c_/*w*_p_ under which the chains spread from single to multiple occupancy in the post arrays of the constant post separation *S*_p_ and of the constant passage width *w*_p_.

	**Circular**			
	*S*_p_ = 12		*w*_p_ = 2	
	*b* = 0	*b* = 20	*b* = 0	*b* = 20
*d*_c_/*w*_p_	5.5	5.5	14.0	14.0
	**Linear**			
	*S*_p_ = 12		*w*_p_ = 2	
	*b* = 0	*b* = 20	*b* = 0	*b* = 20
*d*_c_/*w*_p_	5.5	5.5	9.9	14.0
